# Foam-forming bacteria in activated sludge effectively reduced by rotifers in laboratory- and real-scale wastewater treatment plant experiments

**DOI:** 10.1007/s11356-017-8890-z

**Published:** 2017-04-05

**Authors:** Agnieszka Pajdak-Stós, Wioleta Kocerba-Soroka, Janusz Fyda, Mateusz Sobczyk, Edyta Fiałkowska

**Affiliations:** Institute of Environmental Sciences, Jagiellonian University, Gronostajowa 7, 30-387 Kraków, Poland

**Keywords:** *Lecane* rotifers, Filamentous bacteria, Actinomycetes, Sludge bulking, Wastewater treatment plant

## Abstract

*Lecane inermis* rotifers were shown to diminish sludge bulking due to their ability to ingest the filamentous bacteria in activated sludge. To determine if rotifers are also able to control branched actinomycetes, we investigated three other *Lecane* species (Monogononta). In a week-long experiment, only *Lecane tenuiseta* significantly reduced the density of *Microthrix parvicella* and *Type 0092* filaments, but in a 2-week experiment, actinomycetes were significantly reduced by most of the tested monogonont rotifers: *L. inermis*, *Lecane decipiens* and *Lecane pyriformis*. Rotifers *L. inermis* originating from the mass culture were artificially introduced into real-scale wastewater treatment plant (WWTP) in two series. The WWTP was monitored for 1 year. Rotifer inoculation resulted in diminishing of *M. parvicella* and actinomycete abundance. The experiments showed that different species of rotifers vary in their effectiveness at limiting various types of filamentous organisms. This is the first report demonstrating that one of the most troublesome bacteria, branched actinomycetes, which cause heavy foaming in bioreactors, can be controlled by rotifers*.* Knowledge of the consumers of filamentous bacteria that inhabit activated sludge could help WWTP operators overcome bulking and foaming through environmentally friendly methods.

## Introduction

In most countries with temperate climates, episodes of activated sludge bulking and foaming periodically occur in wastewater treatment plant (WWTP), not only in conventional activated sludge systems but also in membrane bioreactors (MBR) (Cosenza et al. 2013; Di Bella and Torregrossa 2013; Capodici et al. 2015). In properly working activated sludge, various types of filamentous bacteria coexist in amounts that promote the creation of strong flocs and enhance organic matter decomposition (Jenkins et al. 2004). However, when conditions change due to the weather, the type of influent, particular operating conditions or technological problem, the balance can be disrupted, which may result in drastic changes in the amount and proportion of different filamentous bacteria. Their overproliferation leads to activated sludge bulking or foaming or both, and such situation strongly reduces the efficiency of wastewater treatment process. Activated sludge foaming is a common problem worldwide. A foam layer may exceed thickness of 1 m and may cause impairment of foam removal system, worsening of effluent quality, possible dispersal of pathogens in windblown foam and other extreme difficulties in process control (De los Reys III 2010; Pal et al. 2014). Lately yet, another problem caused by high fraction of filamentous and branched bacteria in the activated sludge arose: foaming in anaerobic digesters (Alfaro et al. 2014; Kougias et al. 2014; Subramanian and Pagilla 2015). The occurrence of foaming caused by filamentous bacteria is usually dependent on temperature, and excessive growth of the most troublesome bacteria, *Microthrix parvicella*, is often linked to a decrease in temperature below 15 °C, whereas the growth of foam-forming actinomycetes is usually favoured by temperatures over 15 °C (Eikelboom 2000). However, in a review, Soddel and Seviour (1995) presented conflicting reports about the temperature at which foaming occurs. There were discrepancies between optimal temperatures for nocardioform growth observed in wastewater treatment plants and in pure cultures. Such results suggest that temperature dependence of overproliferation of certain types is not a simple cause-and-effect relation.

Even though there are methods to overcome bulking and foaming, none is free of constraints. One of the proposed solutions is the use of selectors, but this approach is only effective against some types of filamentous bacteria (Jenkins et al. 2004). Currently, plant operators focus on chemical methods, such as the application of metal salts of aluminium or iron or chlorination (Wanner 1994; Jenkins et al. 2004; Pal et al. 2014). As physical and especially chemical methods have their limitation due to evolving resistance of bacteria, also biological methods were recently proposed. One of them is based on the use of specific bacteriophage infecting foam-forming bacteria (Withey et al. 2005; Petrovski et al. 2011; Pal et al. 2014; Liu et al. 2015; Dyson et al. 2016). The other promising biological alternative is application of rotifers that feed on filamentous bacteria. The rotifer *Lecane inermis* has been shown to significantly reduce the abundance of different types of filamentous bacteria: *M. parvicella* (Fiałkowska and Pajdak-Stós 2008), *Nostocoida limicola* (Pajdak-Stós and Fiałkowska 2012), *Type 021 N* (Kocerba-Soroka et al. 2013a), Type 0092 (Drzewicki et al. 2015), *Thiotrix* sp. (Kowalska et al. 2014) and *Haliscomenobacter* (Kowalska et al. 2016).

These results indicate that *L. inermis* rotifer may be considered as a universal filaments consumer, yet so far there were no data about rotifer effectiveness in controlling the branched forms such as actinomycetes producing heavy foaming. Previous direct microscopic observations indicated that actinomycetes were less accessible for *L. inermis* than *M. parvicella*, but no rigorous experiments have been performed to assess the rotifer capability for reducing the actinomycete colonies (Fiałkowska and Pajdak-Stós 2008).

*L. inermis* appears to be a very promising alternative to chemical and physical methods to prevent activated sludge bulking, but this species of rotifer ceases to proliferate at temperatures below 8 °C. The minimum temperature at which *L. inermis* can sustain a positive growth rate is 8 °C, but this has only been observed in one of the four clones of this species (Fiałkowska et al. 2011). The optimal temperature at which this rotifer has exhibited its highest growth rate is 30 °C (Walczyńska et al. 2016), so at lower temperatures, they require relatively old sludge to reach the density at which they can limit the overproliferation of filaments. Additionally, our previous research showed that an initial *L. inermis* density of 100 ind/mL resulted in bulking control (Fiałkowska and Pajdak-Stós 2008). The use of this species in treatment plants relies on introducing the rotifers during a warm season, when they have a good chance of proliferating and quickly reducing the number of filamentous bacteria and thus preventing overproliferation in cold seasons. Only under favourable conditions can they maintain filament density at a proper level (1 or 2 on the Eikelboom scale). However, although rotifers are common constituents of activated sludge communities and can significantly improve the properties of activated sludge by enhancing flocculation, the reduction of suspended solids and excessive sludge, conditions that favour rotifer growth in WWTPs, is poorly recognized. As species differ in their reaction to different environmental factors, it seems reasonable to maintain high rotifer biodiversity in WWTPs and consequently ensure higher resilience of the whole community. Therefore, we must identify other rotifers that are effective in the control of filamentous microorganisms, which was the goal of this work. Through two separate experiments, we investigated the ability of three species of Monogononta rotifers, *Lecane tenuiseta* (Harring, 1914), *Lecane decipiens* (Murray, 1913) and *Lecane pyriformis* (Daday, 1905) to reduce the abundance of *M. parvicella* and Type 0092 and the ability of four monogononts, *L. inermis* (Bryce, 1892), *L. tenuiseta*, *L. decipiens* and *L. pyriformis*, to limit *M. parvicella*, *Sphaerotilus natans* and branched actinomycetes.

## Materials and methods

### Laboratory-scale experiments

#### First experiment

The activated sludge used for this experiment originated from a municipal wastewater treatment plant, coded as Che, located in eastern Poland. The plant eliminates phosphorus and nitrogen and purifies municipal sewage from the single city (about 65,000 inhabitants). At the time of sampling, the filament index was 3.5, according to the Eikelboom scale, with clear dominance of *M. parvicella* (filament index (FI) = 2.0) and Type 0092 as the second most abundant filament (FI = 0.8)*.* The experiment was carried out on tissue culture plates (TPP) kept in darkness at 20 °C. Eight replicates were performed for each species of rotifer, and eight wells without rotifers served as controls. In this first experiment, clones of *L. tenuiseta*, *L. decipiens* and *L. pyriformis* were used, and each clone had been obtained earlier from different samples of activated sludge and maintained under laboratory conditions. At the start of the experiment, 100 individuals of each rotifer species were transferred separately to treatment wells, and during this process, approximately 600 μL of culture medium (Żywiec brand spring water) was also transferred. The same volume of spring water was added to each control well, and afterwards, 1 mL of thoroughly mixed activated sludge was added to each well. After 1 week, three 10-μL samples were taken from each treatment well, and the rotifers were counted. Their density in 1 mL of solution and average population growth rate (*r*) were calculated according to formula *r* = (ln (*n*_t_) − ln (*n*_0_))/*t*, where “*t*” is the day of experiment, “*n*_t_” is the number of rotifers at the end of the experiment and “*n*_0_” is the initial number of rotifers. In addition to these species, solitary native rotifers, such as *L. inermis*, *Cephalodella* and different bdelloid species, were observed, but they were excluded from further analysis as they were present in both the treatment and control wells.

To compare the density of filaments in the treatment and control groups, the density factor of the filamentous bacteria, as described by Kocerba-Soroka et al. (2013a), was analysed. Two 30-μL subsamples were taken from each well, and 22 × 22-mm smears were prepared on microscopic slides. The smears were then Gram stained, and digital images of ten randomly chosen fields of view were taken using a Nikon Eclipse 80i microscope with a total magnification of ×1000. The number of filaments crossing the 85 × 64-μm borders was counted in each image.

#### Second experiment

The second experiment was carried out on activated sludge collected from a small municipal plant, coded as Zel, located near Kraków (south-eastern Poland). The plant purifies sewage from a single village (<1000 inhabitants). The filament index of this sludge was FI = 3.5 with obvious dominance of actinomycetes, and the other filaments observed in the sludge were *M. parvicella* and *S. natans*. The experiment was also carried out on 24-well tissue culture plates (TPP) with eight replicates for each rotifer species and eight control wells. In this experiment, we used four species of *Lecane* rotifers: *L. inermis*, *L. tenuiseta*, *L. decipiens* and *L. pyriformis*, and all of the rotifer clones were cultivated in high-density cultures in the lab. As the rotifers differ in size and growth rate, in this experiment, we decided to transfer 100 μL of well-mixed culture of each clone to wells containing 1 mL of activated sludge with eight replicates for each rotifer species, and 100 μL of Żywiec brand spring water was added to the control wells. Simultaneously, three 100-μL subsamples of original culture were taken and fixed with acidic Lugol solution. Then, all of the individual rotifers were counted, and their initial density per 1 mL was calculated for each species. The culture plates were incubated for 2 weeks in darkness at a constant temperature of 20 °C.

After 1 week and then after 2 weeks, all of the study rotifers were counted, and their population growth rate (*r*) was calculated according to the formula given earlier. To calculate the density factor of the filaments, we modified the methodology described earlier because the morphology of the actinomycetes differs from that of *M. parvicella* or Type 0092*.* Actinomycete filaments are tiny and branched and occur in characteristic colonies, so to obtain comparable results to those of *M. parvicella* or Type 0092, we prepared two smears of 30-μL samples from each treatment and control well and Gram stained them. Each smear was 22 × 22 mm in size, and a Nikon Eclipse 80i microscope with a total magnification of ×1000 was used to count the number of colonies. On each smear, we analysed one randomly chosen band within a 1.826-mm^2^ area between the edges of the smear, on which we counted all actinomycete colonies*.* Thus, two bands were analysed for each treatment well. For the statistical analysis, the average value from two bands per well was calculated. The density of the remaining filaments (including *M. parvicella* and *S. natans*) was also estimated; for the same bands, the filaments crossing their borders were counted.

Analysis of variance (ANOVA with contrasts) was used to detect significant differences in the density of filamentous bacteria between the control and experimental treatments, in which the bacteria remained under pressure from different species of rotifers. Data were transformed (natural logarithm) if they did not meet the assumptions for an ANOVA. The statistical analysis was performed with Statistica 10 (StatSoft Inc. 2011).

### Experiment in real-scale WWTP

In a frame of the project “Integrative system of activated sludge bulking control in wastewater treatment plants”, we inoculated activated sludge in different WWTPs with mass-cultured *L. inermis.* Only one of ten monitored WWTP’s suffers regularly from heavy foaming caused by overproliferation of actinomycetes.

WWTP coded as Zel is a small, conventional, domestic plant without N and P removal system. The volume of aeration tank is 84 m^3^, and the volume of secondary clarifier is 20 m^3^. The WWTP was monitored from December 2014 for over 1 year. Microscopic analyses according to the Eikelboom method (Eikelboom 2000) were performed at 2-week intervals. In this method, the abundance of filamentous organisms observed in a sample is assessed on the base of the reference images included in the method’s manual (Eikelboom) and expressed as degrees on a 0 to 5 scale called the FI. The sum of partial indexes assigned to certain type of bacteria has to be equal to total FI. The densities of proto- and metazoans are expressed as degrees on a scale from 0 to 3 where 0 means none and 3 means numerous cells/colonies per slide. We introduced half-point intervals to the previous scales to attain greater precision.

Mass culture of rotifers *L. inermis* was maintained according to the method described by patent application (Pajdak-Stós et al. 2016b). Generally, the rotifers are kept in plastic vessels in Żywiec brand spring water and feed with the nutrition powder (patent no. EP2993978). In such conditions, they reached abundance about 8000 individuals/mL. Once a week, the rotifers were harvested and in plastic tanks transported to WWTP.

Rotifers were inoculated directly to aeration tank. The dates, physical parameters of activated sludge and data on rotifer culture volume are given in Table 1.

**Table 1:** Time table of rotifer application with parameters measured in aeration tank at the moment of inoculation

Date	Temperature (°C)	pH	DO (mg/L)	Volume (L)
Series I				
04 Feb 2015	9.6	6.6	2.5	11
11 Feb 2015	9.3	6.4	5.4	25
18 Feb 2015	9.1	6.5	0.5	15
25 Feb 2015	8.5	6.7	2.1	10
Series II				
20 May 2015	14.4	6.7	6.9	26
26 May 2015	14.3	6.4	1.3	25
03 Jun 2015	15.4	6.6	1.3	30

Table contains volume of rotifer culture with abundance ca. 8000 individuals/mL

Rotifer abundance and the two foam-forming bacteria: *M. parvicella* and actinomycetes were monitored for 1 year.

## Results

### Laboratory-scale experiments

**Fig. 1:**
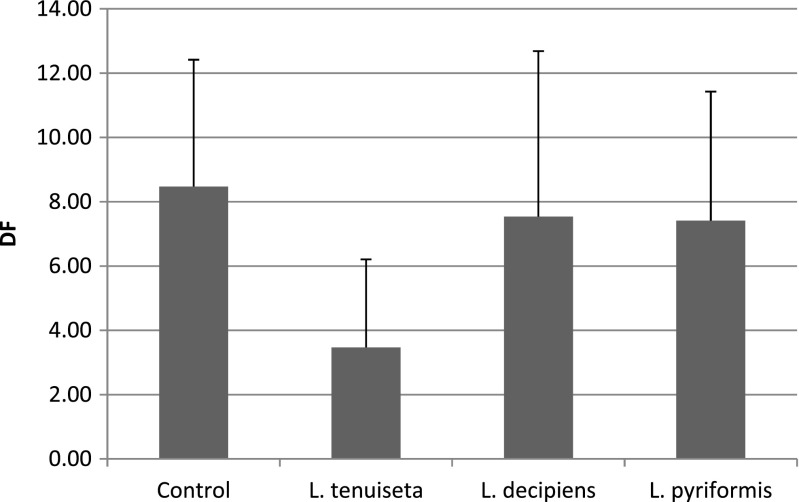
Mean density factor (DF) values for the *M. parvicella* and Type 0092 remaining after 1 week under pressure from different rotifers

**Fig. 2:**
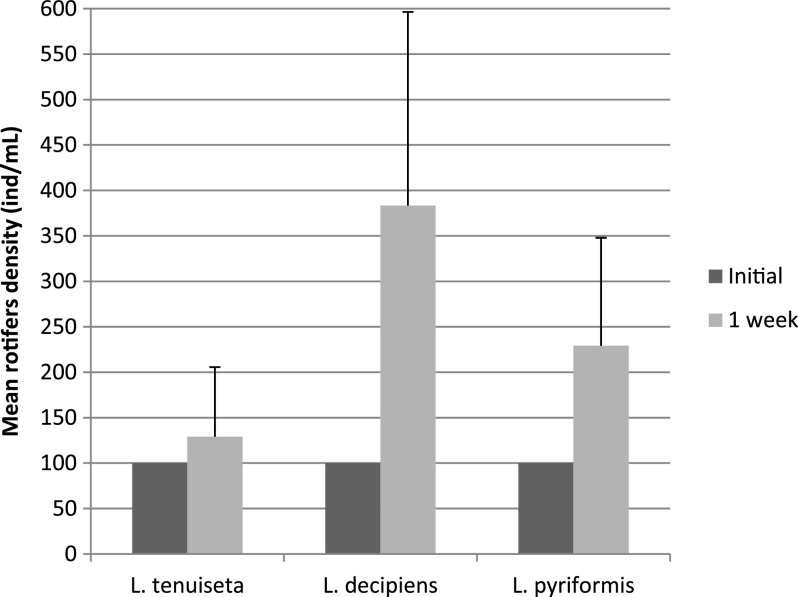
Mean number of rotifers per millilitre after 1 week of experimentation compared to the initial density of 100 ind/mL

Our first experiment showed that only *L. tenuiseta* can significantly reduce the density of filamentous bacteria, such as *M. parvicella* and Type 0092, and compared to the control, the reduction was statistically significant (ANOVA with contrasts, *p* = 0.012). The density of filaments in the treatments with *L. decipiens* and *L. pyriformis* decreased (Fig. 1), but the differences were not significant. Additionally, the number of rotifers increased during the experiment (Fig. 2), and the increase was most pronounced in *L. decipiens* and *L. pyriformis*. The number of the former increased 3.5 times and that of the latter more than 2 times. *L. tenuiseta* also increased in number but at lower rate. Nevertheless, *L. tenuiseta* at a relatively low density significantly reduced the densities of both types of filamentous bacteria.

**Fig. 3:**
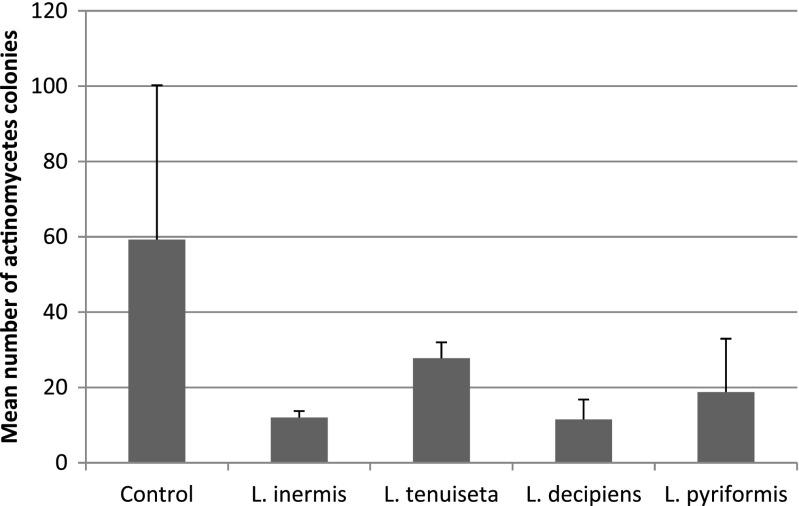
Mean number of actinomycete colonies remaining after 2 weeks under pressure from different rotifer species compared to the controls

**Fig. 4:**
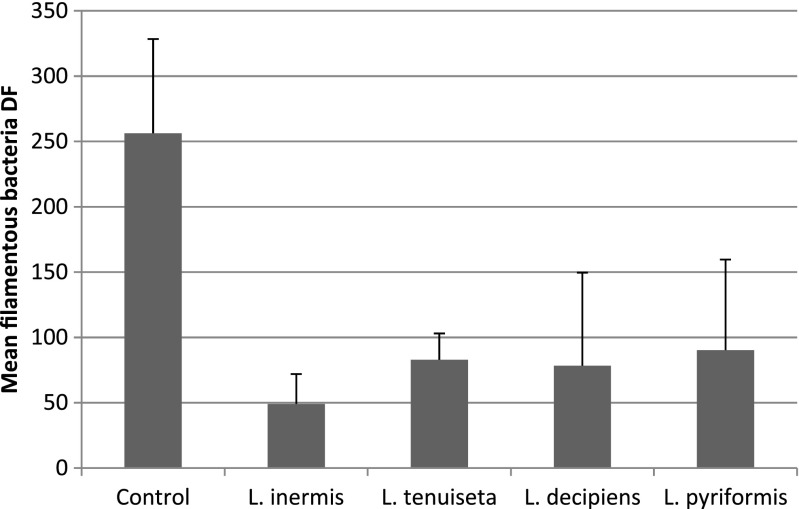
Mean density factor (DF) values for the *M. parvicella* and *S. natans* remaining after 2 weeks under pressure from different rotifers

**Fig. 5:**
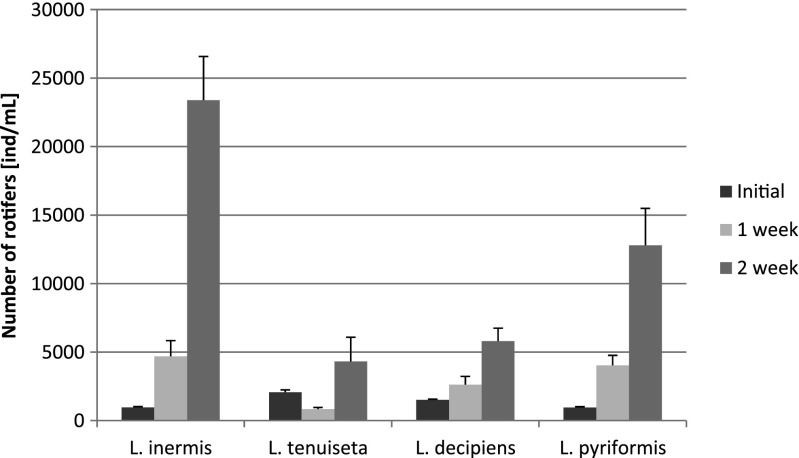
Changes in the mean number of each rotifer species throughout the 2-week experiment

The second experiment indicated that actinomycetes can be significantly reduced by most of the tested rotifer species (ANOVA with contrasts: *L. inermis*, *p* = 0.001; *L. decipiens*, *p* = 0.0004; *L. pyriformis*, *p* = 0.004), but in the case of *L. tenuiseta*, the reduction in the number of actinomycete colonies was noticeable (Fig. 3) but not significant. The densities of the other filaments, *M. parvicella* and *S. natans*, were significantly reduced by all of the tested rotifers (ANOVA with contrasts: *L. inermis*, *p* = 0.0001; *L. decipiens*, *p* = 0.0004; *L. pyriformis*, *p* = 0.0008; *L. tenuiseta*, *p* = 0.0005) (Fig. 4). After 1 week, the number of *L. inermis*, *L. decipiens* and *L. pyriformis* increased 4.8, 1.7 and 4.2 times, respectively, whereas *L. tenuiseta* decreased almost 2.5-fold. After 2 weeks, the density of all of the tested species increased in comparison to the density reached after 1 week. The most abundant was *L. inermis*, whose density increased almost 25-fold within 2 weeks (Fig. 5).

### Experiment in real-scale WWTP

**Fig. 6:**
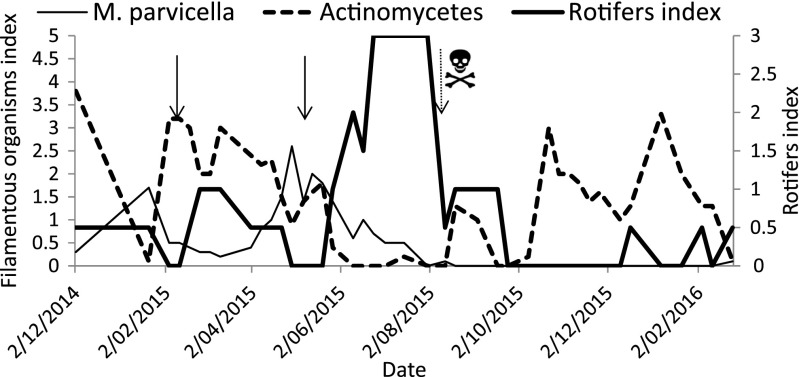
Indexes of rotifers, actinomycetes and *M. parvicella* in activated sludge originating from Zel WWTP. *Black arrows* show the beginning of rotifer inoculation, and *dotted arrow with a scull icon* shows occurrence of predatory fungus in subsamples

The density of rotifers in WWTP and their effect on *M. parvicell*a and actinomycete abundance are shown in Fig. 6. Black arrows point the dates of the start of each series of rotifer inoculation. A month since the first series, their index reached value 1 in a 0 to 3 scale. At the same time, actinomycete abundance visibly decreased. Then, after the following 2 months, rotifers were not visible in the subsamples. Rotifer abundance rapidly increased after the second series of inoculation reaching the highest value in a scale and then in October again dropped to 0 (Fig. 6). Dotted arrow indicated the day of sampling when a predatory fungus *Zoophagus* sp. was spotted in the subsamples. In Fig. 6, repeating pattern of relation between rotifer abundance and both foam-forming bacteria density could be noticed.

## Discussion

Our study showed that all of the tested rotifer species are able to significantly reduce filamentous bacteria and/or actinomycete colonies.

### Laboratory-scale experiments

The first experiment showed that *L. tenuiseta* can effectively reduce the abundance of filamentous bacteria, such as *M. parvicella* and Type 0092, and these results are very promising from the perspective of using this species to prevent activated sludge bulking and as an alternative to *L. inermis*. The morphology of *L. tenuiseta* is very similar to that of *L. inermis*, which is a known consumer of most of the filaments in activated sludge, but *L. tenuiseta* is more adapted to lower temperatures (Fiałkowska et al. 2016). To date, the maximal growth rate reported for *L. tenuiseta* at 8 °C (*r* = 0.16 individuals per day) is much higher than that for *L. inermis* (*r* = 0.03 day^−1^), but *L. inermis* reaches a higher growth rate at 20 °C. According to previous research (Fiałkowska et al. 2011; Fiałkowska et al. 2016), the growth rate of *L. inermis* can be as high as 0.59 day^−1^, whereas the *L. tenuiseta* “*r*” can reach a maximum value of between 0.23 and 0.37 day^−1^. The experiments described in this paper were conducted in pure culture under optimal feeding conditions, but the species-specific growth rate could be modified by external conditions, such as resource competition or the presence of harmful substances. In our first experiment with activated sludge, the growth rate of *L. tenuiseta* reached 0.037 day^−1^, and this value was much lower than the maximum growth rate for this species at 20 °C as well as in comparison with the two other *Lecane* study species. In the case of *L. decipiens* and *L. pyriformis*, the *r* value reached 0.192 and 0.118 day^−1^, respectively. As activated sludge is a mixture of different, often competing species subjected to a combination of typically unknown substances, we could only speculate as to why the growth of *L. tenuiseta* was inhibited. We could not exclude the possibility that *L. tenuiseta* is more vulnerable to toxic substances than the other tested *Lecane* species. Additionally, *L. inermis* and *L. tenuiseta* are characterized by similar dietary preferences and could potentially inhabit the same niche, but *L. tenuiseta* proliferates more rapidly at lower temperatures compared to *L. inermis*. Different temperature preferences are very advantageous as means for these species to minimize competition for resources, and seasonal temperature fluctuations seem to favour the coexistence of these two species at intermediate temperatures and the dominance of one or the other at extreme temperatures. Edmondson (1946) indicated that temperature and food availability are the most important factors affecting the lifespan, population abundance and growth rate of rotifers, and previous research (Fiałkowska et al. 2016) showed that the growth rate of *L. tenuiseta* at 20 °C ranges from 0.10 to 0.41 day^−1^ depending on the clone or linage. Our experiments were conducted at 20 ± 1 °C, at which the growth rate of *L. tenuiseta* was the lowest (Fig. 2) compared to the other tested rotifers. Despite this result, *L. tenuiseta* was, surprisingly, the most effective of the study rotifers at reducing filamentous bacteria in the first experiment. Although the increase in abundance was highest for *L. decipiens* and *L. pyriformis*, almost 3.5 and 2 times, respectively, they did not significantly reduce any of the investigated filaments. In the second experiment, on the other hand, these two species were able to reduce *M. parvicella.* The probable reason for this effect is the prolonged exposure of the filamentous bacteria to the rotifers; the second experiment lasted for 2 weeks, during which *L. decipiens* and *L. pyriformis* significantly reduced the density of filamentous bacteria. Little information on the biology of these species is available, but these rotifers have a wide tolerance to temperature. *L. decipiens* occurs within a temperature range from 3 to up to 25 °C but reaches maximum abundance at approximately 16 °C (Bērzinš and Pejler 1989). There have been no earlier reports on the occurrence of *L. decipiens* and *L. pyriformis* in activated sludge.

The results of the second experiment also demonstrated that *L. inermis*, *L. decipiens* and *L. pyriformis* significantly limited the number of actinomycete colonies, and these organisms are troublesome due to their morphology. Although they are usually classified as filamentous organisms, they grow in “bushy” colonies with branched filaments, and during their excessive growth, which usually occurs in warm season, this type of filament creates a dense foam on the activated sludge surface and negatively influences its sedimentation properties. For the first time, our results showed that the density of branched actinomycetes can be significantly reduced by *L. inermis*, *L. decipiens* and *L. pyriformis* (Fig. 3)*.* However, although the mean density of the actinomycete colonies decreased in the presence of *L. tenuiseta* (Fig. 3), the effect was not significantly different than the control. This filament also seemed to be affected by *L. tenuiseta*, but the effect was too weak to be significant relative to the highly variable control. There are several possible explanations of this effect. First of all, the activated sludge used in this experiment might be harmful to this rotifer (Fig. 5) due to the presence of toxins in the inflowing sewage, which was indicated by the decreasing number of *L. tenuiseta* after 1 week compared to its initial density. Interestingly, a similar pattern was observed in the first experiment, so we could not exclude that *L. tenuiseta* is the most vulnerable species. Fortunately, as can be seen in Fig. 5, this species is able to adapt and improve its ability to proliferate. Another explanation could be related to the food preferences of this species; although actinomycetes were dominant in the experimental sludge, there were also plenty of non-branched filamentous bacteria that were significantly reduced by *L. tenuiseta*.

The feeding behaviours of the species used in our experiment have not been examined. However, some information about grazing by rotifers on filaments in activated sludge appeared several years before and only concerned one *Lecane* species. Therefore, we cannot exclude the possibility that there are some differences in the construction of the feeding apparatus of *L. tenuiseta* that makes it less effective at feeding on filaments, especially branched ones such as those of the actinomycetes*.*

### Experiment in real-scale WWTP

Our results evidenced that artificial inoculation of rotifers *L. inermis* is possible in real-scale WWTP and that higher abundance of rotifers results in effective control of foam-foaming bacteria such as *M. parvicella* and actinomycetes. Although there are reports on negative correlation between rotifers and filament index in real-scale WWTPs (Fiałkowska and Pajdak-Stós 2008), this is the first observation of rotifer impact on branched foam-foaming actinomycetes. In Fig. 6, it is clearly depicted that the second series of rotifer inoculation led, after temporary decline, to rapid proliferation of artificially introduced rotifers in aeration tank. Increasing density of rotifers was followed by the decrease in the density of *M. parvicella* and actinomycetes. As Fig. 6 shows, the first inoculation was less successful probably due to the temperature too low to ensure high enough rotifer growth rate. This is consistent with our earlier results (Fiałkowska et al. 2011; Pajdak-Stós and Fiałkowska 2012) showing that population growth rate of *L. inermis* at 8 °C is close to 0. Temperature in aeration tank during the first series of inoculation varied between 8.5 and 9.5 °C. Rotifer abundance a month later appeared to be high enough to reduce actinomycete index by half within less than 4 weeks (Fig. 6). Second series of inoculation conducted when temperature varied between 14.3 and 15.4 °C resulted in relatively rapid rotifer proliferation and, in consequence, reduction of *M. parvicella* and actinomycete abundance. The reason why rotifers were not observed in August was probably massive occurrence of predatory fungus *Zoophagus* sp. earlier reported as a threat to rotifer population in WWTPs (Pajdak-Stós et al. 2016a).

Most importantly, this research found that species other than *L. inermis* are capable of limiting the growth of filamentous bacteria and, especially, branched actinomycetes. This knowledge will be used to improve biological methods of bulking control, and a higher biodiversity of artificially introduced rotifers could help to maintain the resilience of the rotifer community in the fluctuating WWTP environment. Using various species of rotifers as natural consumers of filamentous bacteria could be an alternative method to control the most troublesome bacteria and protect treatment plants from bulking throughout the year. Additional advantage of described biological method is that, as it was shown, earlier increasing density of rotifers does not negatively influence physicochemical parameters of effluent (Kocerba-Soroka et al. 2013b). Moreover, rotifers play an important role in floc formation, reduction of excess biomass production and consumption of dispersed bacteria in activated sludge (Lapinski and Tunnacliffe 2003).

Our results show for the first time how effective artificially introduced rotifers could be in controlling *M. parvicella* and actinomycetes—bacteria causing heavy foaming in WWTPs. The monitoring of rotifer abundance with relation to other process parameters in real-scale WWTP helped to define optimal condition for rotifer inoculation on the one hand and potential limitation of the method on the other.

## Conclusions

*Lecane* rotifers are able to control abundance of branched actinomycetes, one of the main causes of scum formation in WWTPs. Our research showed that rotifer species: *L. inermis*, *L. decipiens*, *L. pyriformis* and *L. tenuiseta* are all able to ingest filamentous and branched bacteria and could be used as a promising biological tool for control of activated sludge bulking and foaming. The knowledge gained in the course of experiments, especially in real-scale WWTP, will help WWTP operators to optimize sludge sedimentation properties.
